# The Embodied Hijack: when Pleistocene minds meet disembodied artificial intelligence

**DOI:** 10.3389/fpsyg.2026.1889386

**Published:** 2026-08-10

**Authors:** Sheila L. Macrine

**Affiliations:** 1 Department of Education, University of Massachusetts Dartmouth, North Dartmouth, MA, United States; 2 Active Inference Institute Inc., Davis, CA, United States

**Keywords:** active inference, agency attribution, anthropomorphism, embodied cognition, embodied hijack, epistemic alignment, evolutionary mismatch, large language models

## Abstract

The rapid integration of artificial intelligence into everyday life has intensified a long-standing feature of human cognition: the attribution of agency, intention, and understanding to nonhuman systems. People describe language models, virtual assistants, and autonomous technologies as if these systems know, decide, want, or understand, and they continue to do so even when they know the systems have no inner life. The standard account dismisses this as naïve anthropomorphism, the misfiring of evolved agency-detection systems calibrated in the Environment of Evolutionary Adaptedness. We argue that the standard account is incomplete. It explains the immediacy of anthropomorphic response but not its persistence even when users know the system has no mind. Drawing on evolutionary psychology, philosophy of agency, and the active inference framework, we advance the Embodied Hijack hypothesis. Across evolutionary time, fluent communication and contingent responsiveness were produced only by embodied, self-maintaining agents with vulnerability and temporal continuity. Current conversational LLM deployments are the first class of entity to reproduce these signals without the grounding properties — biological self-maintenance, vulnerability, and temporal continuity — that historically produced them. The result is a predictable misalignment: users’ inferential systems treat these signals as evidence of agency they were calibrated to indicate, producing systematic misattribution. The Embodied Hijack is not irrationality. It is the optimal predictive response of a Pleistocene-calibrated brain to the rupture of the evolutionary invariant that once tied fluent communication to embodied self-maintenance. The framework yields a unique empirical signature: anthropomorphic response will track the signal profile of a system independently of users’ propositional beliefs about what the system is. We close by arguing that the goal is epistemic alignment — bringing how users interpret these systems into correspondence with what these systems actually are — and that this alignment is achieved through interface design rather than user education.

## Introduction

The rapid integration of artificial intelligence into everyday life has intensified a long-standing feature of human cognition: our tendency to attribute agency, intention, and understanding to nonhuman systems (Epley et al., 2007; Reeves and Nass, 1996; Thellman et al., 2022). Recent work argues that this phenomenon has now shifted in character. Today’s contemporary large language models exhibit such convincingly human-like communicative abilities that the anthropomorphic qualities no longer reside solely in users’ projections, but also appear to be present in the systems themselves (Peter et al., 2025). People often describe language models, virtual assistants, and autonomous technologies as if they know, decide, want, or understand. This tendency persists even when users explicitly know that these systems do not have minds or consciousness, a pattern consistently observed across studies of human interaction with artificial agents (Waytz et al., 2010a; Reinecke et al., 2025).

The pattern is recognizable in everyday interactions, but it is not all of one kind. Some practices reflect convenience or social habit: users routinely add “please” and “thank you” to prompts even though the system cannot register politeness (Nass and Moon, 2000; Reeves and Nass, 1996). Others suggest something more — softening refusals as if maintaining a relationship, apologizing to a code assistant when their own instructions were unclear, feeling reassured when a chatbot “listens,” or chastened when it pushes back. These deeper responses are not confined to inexperienced users, and they persist after users are told, and accept, that the system has no inner life. The Embodied Hijack hypothesis targets this latter pattern: cases where users’ felt social presence, trust calibration, or in-the-moment mental-state attribution exceeds their explicit understanding of the system’s non-agentive organization. Pragmatic shorthand and social-script behaviors are also worth studying, and dispositional and motivational differences shape how readily individuals extend mind to nonhuman targets (Epley et al., 2008; Waytz et al., 2010a), but these are not the focus here. Any account of how human cognition meets contemporary AI must explain why the deeper responses are so difficult to switch off.

This phenomenon is often dismissed as naïve anthropomorphism. The term refers to the attribution of human mental states, intentions, and characteristics to nonhuman entities (Epley et al., 2007). The standard framing treats it as a cognitive bias or misfiring of evolved agency-detection systems calibrated in the Environment of Evolutionary Adaptedness (EEA; Barrett, 2000; Cosmides and Tooby, 1992; Guthrie, 1993). We use *calibrated* here in its evolutionary-cognitive sense: cognitive systems are tuned by selection to environmental regularities such that their inferences are reliable when those regularities hold. For example, the machinery that evolved to detect agents in ancestral environments is now triggered by fluent artificial systems. These systems reproduce the surface signals of agency — linguistic fluency, contingent responsiveness, conversational coherence — without the underlying organizational properties that historically produced them. The signals these systems produce historically indicated what Ramstead et al. (2020) and Hohwy (2016, 2026) call self-evidencing organization. That is, systems whose actions function to confirm their own generative models (internal representations of how the world produces sensory input) and maintain their existence. LLMs possess the signals without the organization. The full account of this distinction is developed in later sections.

Understanding why this misfiring occurs requires looking at the conditions under which agency detection evolved. The EEA, a concept originally introduced by Bowlby (1969), refers to the set of recurring selection pressures that shaped human cognitive adaptations over evolutionary time. It is not a specific place or historical period, but a statistical composite of ancestral conditions under which psychological mechanisms evolved (Bennett, 2018; Tooby and Cosmides, 1992). For humans, these conditions largely consisted of small-scale, socially dense Pleistocene hunter-gatherer environments, where survival depended on continuous interaction with other embodied agents.

Within these environments, agency attribution was not optional. It was a core adaptive capacity shaped under conditions in which failing to detect a real agent could carry severe costs, while over-attributing agency was comparatively low risk. As a result, human cognitive systems developed strong priors that favor the detection of agency, particularly under conditions of uncertainty.

When these evolved priors encounter fluent artificial systems, the standard evolutionary-psychology account explains the resulting anthropomorphism as the misfiring of those priors. We argue that this account is incomplete. It explains why anthropomorphism occurs initially, but not why it persists even when people know the system has no mind, nor why it grows stronger as systems become more capable (Vafa et al., 2024; Waytz et al., 2010b). The deeper structural tension is a Pleistocene mismatch (Li et al., 2018; van Vugt et al., 2024) between evolved predictive systems and the organizational architectures of modern artificial agents. Human ancestors evolved over roughly two million years in a world where fluent communication, contingent responsiveness, and context-sensitive behavior were reliable indicators of embodied, self-maintaining organisms (Clark, 2008; Tomasello, 2014; Varela et al., 1991). Every signal that cued agency was produced by a body with metabolic stakes. Current conversational LLMs are the first class of entity to reproduce those signals without the corresponding organizational properties of self-maintenance, vulnerability, and temporal continuity. This produces a failure of predictive alignment between evolved inference and the entities it encounters (Friston, 2010; Parr et al., 2022; Quattrociocchi et al., 2025).

We term this response the Embodied Hijack: the hypothesis that disembodied systems produce human-like communicative signals that lead people to interpret them as intentional agents. The result is systematic misattribution by the user when those signals are decoupled from the embodied, self-maintaining systems that historically produced them. Anthropomorphism, in this framing, is not simply a cognitive bias but a context-sensitive inferential strategy, one that tracks the conditions under which a cue is reliable and fails systematically when those conditions no longer hold.

What distinguishes the Embodied Hijack from existing accounts of anthropomorphism, evolutionary mismatch, and predictive processing is twofold. First, we identify the historically stable coupling between signals of agency and embodied, self-maintaining organization as an evolutionary invariant — a regularity that calibrated human predictive systems across the entire window in which social cognition was shaped. Existing accounts identify mismatch but do not characterize this specific coupling as the structural feature being violated. Second, the framework yields a prediction that follows from this characterization and not from existing accounts: anthropomorphic responses will track the signal profile of a system independently of users’ propositional beliefs and independently of system novelty or expectation effects. Evolutionary mismatch accounts predict over-attribution under uncertainty; predictive processing accounts predict that priors will resist disconfirmation; existing anthropomorphism accounts predict reliance on cues like fluency or contingency. None of these alone predict that the magnitude of anthropomorphic response will scale specifically with the degree to which a system reproduces the full signal profile that historically indicated embodied self-maintenance, while remaining dissociable from what users explicitly know about the system. This is the empirical signature the framework targets.

Throughout this paper, agency is understood as an emergent organizational pattern rather than a unitary property: how a system generates goals, models and solves problems, and couples with its environment. For the purposes of this paper, we treat agency as a spectrum of organizational properties across systems rather than a binary classification (Macrine et al., 2026; Levin, 2022). Critically, goal generation and intelligence vary independently. Large language models exhibit high intelligence with absent goal generation, while *C. elegans* (a small, transparent, free-living nematode worm; Levin and Martyniuk, 2018) exhibits endogenous goal generation with minimal intelligence. The present argument centers on the dissociation between generative modeling, which contemporary artificial systems exhibit at scale, and embodied system-environment coupling, which they lack.

To develop this claim, we integrate four lines of work. Evolutionary accounts of agency attribution, including early modular formulations (Fodor, 1983; Tooby and Cosmides, 1990, 1992), explain why agency detection is biased toward over-attribution under uncertainty. The present paper develops this through predictive processing rather than a modular architecture. Predictive processing holds that the brain actively generates and updates predictions rather than passively receiving input (Clark, 2013, 2015). Philosophical accounts of the intentional stance and shared intentionality (Dennett, 1987; Searle, 1980; Tomasello, 2014) show that agency attribution is often rational under ordinary conditions but depends on embodied coordination to remain stable. The active inference framework (Friston, 2010; Parr et al., 2022) models cognition as the embodied minimization of prediction error, grounding agency in self-maintaining organization rather than abstract computation. Finally, the ecosystems of intelligence framework (Friston et al., 2024) provides a multi-scale account of how biological and artificial systems can participate in nested, interacting forms of intelligence without reducing them to a simple agent versus non-agent dichotomy. We close with implications that follow from this account for: (1) how intelligence should be conceptualized when behavioral signals of agency can be decoupled from the embodied, self-maintaining organization that historically produced them; (2) why user misunderstanding of LLM cognition persists despite explicit knowledge; and (3) how AI systems can be designed so that users’ interpretive responses align with the organizational properties of the systems.

Together, these implications converge on a single goal: epistemic alignment — bringing how users interpret these systems into correspondence with what these systems actually are. The term is used in adjacent literatures on AI safety and human-computer interaction, where it concerns the calibration of user trust and reliance to actual system capability (Vasconcelos et al., 2023). We use it here in a related but distinct sense: not the calibration of trust to capability, but the alignment of users’ interpretive categories — what users perceive a system to be — with the underlying organization of the system producing that interpretation.

## The Pleistocene legacy

This section revisits evolutionary accounts of agency attribution, not as a general review, but to identify the environmental regularities that shaped human expectations about agents. These accounts explain why humans readily attribute agency under uncertainty. However, they also reveal a deeper constraint: the conditions under which such attributions were historically reliable and that contemporary artificial systems now place under strain.

## The evolution of agency attribution

To see why the standard account is incomplete, we first need to see what the standard account claims. Modern evolutionary accounts of cognitive architecture are often traced to Fodor’s The Modularity of Mind (1983). Fodor proposed that certain aspects of cognition operate through domain-specific, fast, and automatic subsystems tuned to recurrent classes of ecologically relevant input (Barsalou, 2008; Tooby and Cosmides, 1990) extended this approach into the thesis of massive modularity. As they argue, the human mind comprises many evolved mechanisms specialized for recurrent adaptive problems, including the detection of agents and the inference of intentions. While these modular accounts have been highly influential, they have also been challenged by the embodied turn in cognitive science, often grouped under the umbrella of 4E cognitive science, which holds that thinking is not brain-bound but is Embodied (involves the whole body), Embedded (situated in the environment), Enacted (arises from action), and Extended (utilizes external tools and environment) (Anderson, 2010; Barsalou, 2008; Clark, 2008; Gallagher, 2017; Macrine and Fugate, 2020, 2022; Newen et al., 2018; Varela et al., 1991). These approaches treat cognition not as a set of internal processes operating over abstract representations, but as a dynamic activity distributed across brain, body, and world, in which perception, action, and environmental structure are mutually constitutive. Agency attribution, accordingly, is not the product of a discrete cognitive function but an inferential process that emerges from how embodied systems engage with and interpret their environments.

Within the evolutionary-psychology tradition, Error Management Theory (Haselton and Buss, 2000) emphasizes that inference under uncertainty is shaped by asymmetric costs. In ancestral environments, the failure to detect a real threat, a false-negative error, often carried catastrophic fitness costs, including death, injury, or the loss of offspring. By contrast, mistakenly identifying a harmless stimulus as dangerous, a false-positive error, typically incurred only minor, recoverable costs, such as a brief startle response, wasted energy from fleeing, or momentary vigilance (Haselton et al., 2006). Given this asymmetry in error costs, natural selection favored psychological mechanisms biased toward over-detection and heightened caution. This logic, often termed the “smoke detector principle” in evolutionary psychology and medicine, holds that it is adaptive to generate many false alarms rather than risk missing a single real threat, such as a snake or hidden predator (Nesse, 2001, 2005). The same logic is reflected in accounts of hyperactive agency detection, in which ambiguous stimuli are preferentially interpreted as agents (Barrett, 2000, 2004; Fields, 2014; Guthrie, 1993). This logic applies directly to threat detection involving dangerous animals, including predators and venomous creatures that posed recurrent risks throughout our evolutionary history.

This evolutionary logic promotes the formation of perceptual and inferential priors that systematically lower the threshold for detecting agency in ambiguous or uncertain environments. More recent theoretical accounts grounded in predictive processing suggest that such biases emerge not from a dedicated module, but from the brain’s general practice of minimizing prediction error through Bayesian inference (a statistical approach in which prior expectations are updated in proportion to the strength of incoming evidence) (Clark, 2013, 2015; Da Costa et al., 2021; Friston, 2009, 2010). As Clark (2015) develops the view, the brain operates as an active prediction engine rather than a passive receiver of sensory input, continually generating predictions about incoming data and updating them against the errors between expectation and reality. This framework allows organisms to navigate uncertain and dynamic environments efficiently (Orlandi and Lee, 2019). Clark (2008) situates this within a broader embodied view of cognition, in which cognition involves loops that “promiscuously criss-cross the boundaries of brain, body and world” (p. xxviii). Because agents, including predators, prey, and fellow humans, were highly relevant to ancestral fitness, humans develop strong priors expecting agency in the environment (Maij and Van Elk, 2019). When sensory input is noisy, incomplete, or ambiguous, these high priors exert strong top-down influence, leading the system to interpret ambiguous stimuli, such as rustling sounds, unexplained movements, or patterns in noise, as intentional agents. The result is a systematic bias toward over-attribution of agency that arises mechanistically from expectation-driven perception (Andersen, 2019; Atkinson, 2024; Friston, 2012).

Related capacities, including theory of mind (Baron-Cohen, 1995; Wellman, 1990) and specialized perceptual systems such as face detection (Kanwisher et al., 1997), are typically understood as components of this evolved social-cognitive architecture.

Applied to artificial intelligence, the evolutionary-psychology account suggests a straightforward explanation. Systems that exhibit linguistic fluency and contingent responsiveness trigger these evolved mechanisms, leading to anthropomorphic interpretation. For this reason, such responses reflect a bias that, in principle, could be corrected through education or improved understanding.

We argue that the problem is not a simple misfiring of evolved detection mechanisms, but a mismatch between evolved expectations about agents and the structure of contemporary artificial systems.

### The evolutionary invariant

What the standard evolutionary account understates is the stability of the environment in which agency attribution evolved. Across evolutionary time, communicative fluency, contingent responsiveness, and norm-sensitive behavior were not merely correlated with embodied agents. They were produced by them. That is, every instance of fluent, context-sensitive communication in ancestral environments came from a living body with metabolic stakes, and there was no other way for such signals to be generated. This held across species as well as within humans. For example, alarm calls from birds, vocalizations from primates, and warning signals from other animals all originated from embodied organisms whose communicative behavior was grounded in their own survival pressures. Developmental evidence reinforces this pattern. Even preverbal infants treat the capacity for contingent, turn-taking communication as a primary cue for agency, attributing agent-status to otherwise non-humanlike entities when those entities display conversational structure, but not when they lack it (Beier and Carey, 2014; Reinecke et al., 2025). However, limited forms of signal decoupling did exist. For example, a deceiver could produce false words while concealing true intentions. Indirect signaling, such as alarm calls referring to distant or unseen predators, could also convey information about absent agents. But these exceptions confirm rather than undermine the invariant. A deceiver is still an embodied agent. An alarm call still originates from a biological organism with survival stakes. Wind, water, dreams, and the perception of agency in weather and natural events all pervade animist worldviews and can elicit agency attributions (cf. Guthrie, 1993). Cultural and symbolic forms of mediated agency present a more sophisticated case. Humans have long interacted with masks, puppets, theatrical performers, religious icons, spirit mediums, fictional characters, writing, and recorded voices — practices that decouple some agency-signals from their embodied sources. These cases are real and important. They show that humans can engage with mediated or symbolic agency under structured conditions. But they remain partial decouplings: a masked performer is still an embodied agent; a puppet is animated by one; recorded voices play back the signals of past embodied speakers; writing presents the residue of a writer; religious icons function within ritual contexts where embodied participants supply the interactional reciprocity; fictional characters require a reader to supply the imagined exchange. None reproduce the full signal profile that the Embodied Hijack engages: sustained, contingent, real-time, norm-responsive interaction at human conversational pace, generated continuously by a non-embodied source. What ancestral environments never contained was a non-embodied source of sustained fluent communication, conversational coherence, and norm-sensitive interactional behavior. A system exhibiting these signals was always a biological organism embedded in processes of metabolism, vulnerability, and survival (Laland and Brown, 2011; Tooby and Cosmides, 1992).

We refer to this regularity as the evolutionary invariant: the historically stable coupling between signals of agency and the embodied, self-maintaining systems that generated them. The relationship is invariant in the technical sense that it held across the entire window in which human social cognition was calibrated, with no class of exception sufficient to weaken the underlying expectation. What human cognition tracked was not the signals themselves, but the systems behind them — and because the coupling was reliable, tracking the signals was equivalent to tracking the systems. This invariant structured the expectations of human cognitive systems, ensuring that signals such as fluent communication reliably indicated the presence of an agent with stakes in the world. To our knowledge, this coupling has not previously been characterized as a historically stable regularity under which agency attribution evolved. The closest existing accounts come from two literatures. Embodied cognition identifies the grounding of communication in bodily processes (Clark, 2008; Varela et al., 1991), and recent work on LLM anthropomorphism notes the mismatch between evolved expectations and artificial systems (Shanahan et al., 2023). Neither, however, frames this as a specific invariant that structured the calibration of human predictive systems across evolutionary time.

These systems shared a common organizational profile: self-maintenance, vulnerability, and temporally integrated continuity.

#### Self-maintenance

Living systems continuously sustain and reproduce their own organization in the face of entropy. Maturana and Varela (1980) describe this as autopoiesis, the capacity of a system to maintain itself from within.

#### Vulnerability

Biological agents are subject to damage, depletion, and death. This vulnerability grounds meaningful action and underwrites what Dennett (1987) characterizes as the conditions for treating a system as an intentional agent.

#### Temporally integrated continuity

Agents persist over time not merely as continuous entities, but as temporally extended systems whose actions and commitments unfold across multiple timescales. As Ferrero (2022b) argues, agency depends on the coordination of temporally local perspectives into a unified structure of action. Individual actions are situated within ongoing trajectories of intention, allowing agents to sustain commitments, maintain goals, and remain accountable across time.

These properties do not operate independently. Instead, they work together as an integrated organizational structure. Based on findings from comparative cognition and evolutionary biology, this structure is shared by all systems known to produce the signal profile that leads humans to attribute agency (Laland and Brown, 2011; Tomasello, 2014). Within this evolutionary record, communicative signals were grounded in embodied, temporally extended processes of self-maintenance. The claim is not that no exceptions are conceivable, but that the historical record does not contain them at the scale or in the form that would have shifted the calibration of human inference.

This perspective is consistent with evolutionary accounts that emphasize the co-constitution of cognition, body, and environment, in which psychological adaptations cannot be understood independently of the ecological and embodied systems in which they evolved (Barrett, 2000).

## The emerging tension

This invariant now stands in tension with contemporary systems. Signals that were once reliably coupled to embodied, self-maintaining agents now appear in systems that do not share those properties. The result is a condition in which established cues for agency continue to guide inference, even as their organizational basis becomes increasingly ambiguous.

Anthropomorphic responses, in this context, are not easily overridden by explicit knowledge. Informing users that a system lacks understanding does not eliminate the inferential pull of signals that, over evolutionary time, were consistently reliable. Rather than a belief that can simply be revised, the expectation that fluent communication reflects an underlying agent is a feature of cognitive systems shaped under conditions of long-standing regularity.

Explaining this pattern requires moving beyond accounts that frame it primarily as a form of bias. What is needed instead is a framework that captures how predictive systems respond when long-standing regularities are disrupted. The following section turns to philosophical accounts of agency and embodiment to clarify the conditions under which agency attribution remains stable.

## Philosophy of agency and the embodied turn

The evolutionary account explains why humans are biased toward detecting agency under uncertainty. It does not by itself settle two distinct questions that philosophical and cognitive science traditions address in turn. The first is conceptual: what does agency actually consist in, and what are the limits of accounts that identify it with behavioral output? The second is normative: under what conditions is attributing agency to a system a rational inferential strategy, and what are the limits of that strategy?

### What agency is: beyond behavior

If evolutionary accounts explain why humans readily attribute agency, philosophical and cognitive science traditions clarify what agency consists in and why it cannot be reduced to surface behavior. Across these traditions, a consistent insight emerges. Agency is not defined solely by what a system does, but by how its capacities are organized and sustained over time (Ferrero, 2022a).

Philosophical accounts of agency have long emphasized the distinction between behavior and the underlying structures that make behavior meaningful. Searle (1980) argued that the production of appropriate outputs is not sufficient for understanding, distinguishing between systems that merely simulate cognition and those that possess intrinsic intentionality. His Chinese Room argument is the canonical statement of this view: syntactic competence, however sophisticated, does not constitute semantic understanding. Dennett (1987), in contrast, showed that treating a system as an intentional agent can be a useful predictive strategy when its behavior exhibits sufficient coherence. Together, these positions mark a tension central to the present argument. Behavior can justify the attribution of agency, but whether the attribution is accurate depends on the organization of the system producing it. We share Searle’s emphasis on organization. Organizational properties, not behavioral output alone, distinguish different forms of cognitive system.

This tension becomes sharper in embodied, embedded, and enactive approaches to cognition. These traditions reject the idea that cognition can be understood as internal, brain-bound computation. Instead, cognition is treated as an activity that unfolds through the dynamic coupling of brain, body, and environment (Clark, 2008; Gallagher, 2017; Macrine and Fugate, 2020, 2022; Varela et al., 1991). Perception and action are not separable stages, but mutually constitutive processes, and cognitive capacities emerge through ongoing interaction with the world.

The phenomenological tradition makes this point still sharper. For Merleau-Ponty (1945/2012), experience is not something that happens inside a body and then represents an external world; it is the activity of a body engaged with a world. The perceiving, intending, acting self is constituted through this engagement, not behind it. Read this way, the embodied self is not an optional layer added to an information-processing core, but the very condition under which agency, perception, and meaning take on their structure. A system without a body that can be hungry, oriented, fatigued, or at risk has no perspective from which the world could come to matter in this way.

Within this shift, agency is increasingly understood as grounded in the organizational properties of living systems. Biological agents are not passive processors of information. They are self-maintaining systems that regulate their own states in the face of environmental perturbation. Their behavior is structured by the need to preserve viability, giving rise to goal-directed action that is intrinsically tied to survival and persistence. This view aligns with work on autopoiesis (Maturana and Varela, 1980) and with contemporary formulations in active inference, where cognition is modeled as the minimization of prediction error in systems that must maintain themselves over time (Friston, 2010; Parr et al., 2022; Pezzulo et al., 2026).

From this standpoint, agency cannot be reduced to input-output mappings or behavioral fluency alone. It depends on a deeper organizational structure in which perception, action, and regulation are integrated within a system that has something at stake in its continued existence. Agency is therefore not merely a matter of producing appropriate responses, but of doing so as part of an ongoing process of self-maintenance and temporal continuity.

Recent work across disciplines reinforces that agency is not a single property but a structured set of capacities operating across multiple dimensions. Ferrero (2022a) identified four capacities across disciplines. In robotics and AI, agency usually means the capacity to initiate a causal chain — to make something happen. In biology, especially in work on autopoiesis, it means something closer to self-constitution: the capacity of a system to sustain its own identity over time by producing and maintaining itself. In cognitive science, agency tends to mean psychological causation: acting from internal representations, beliefs, and goals. And in philosophy, particularly in normative theory, agency means reason-responsiveness: acting in accordance with norms and reasons one can be held to.

These are not different definitions of the same thing. They are different capacities, identified across different disciplines, and treating them as a single property is precisely what the standard framing of agency tends to do. Ferrero’s framework is useful here because it lets us see these capacities separately — and once we do, the question of which capacities a given system actually possesses becomes a question we can ask, rather than one the word *agency* settles in advance.

For example, a bacterium initiates action and sustains its identity through autopoiesis, but operates without norms or internal representations in any rich sense. Animals act and self-maintain, but most lack the normative responsiveness and reflective self-governance that characterize human agency. What behavioral fluency in a large language model reproduces is the surface of agentive interaction; what it leaves entirely absent is the embodied, self-maintaining organization that historically generated such signals.

This pluralist treatment also clarifies what large language models do and do not possess relative to different forms of agency. The present paper does not deny that LLMs exhibit some agency-relevant capacities; what it denies is that they possess the form of agency the Embodied Hijack engages, namely the biological self-maintenance underwritten by the evolutionary invariant. Table 1 maps five forms of agency, drawn from Ferrero (2022a) and adjacent literatures, against what current conversational LLMs possess, simulate, or lack. The two-dimensional structure of mind perception identified by Gray et al. (2007), in which Agency (capacity to plan, decide, and act) and Experience (capacity to feel) emerge as separable factors, provides empirical support for treating these forms as distinct rather than interchangeable.

**TABLE 1:** Forms of agency and the relationship of current conversational large language models to each.

Form of agency	What it is	LLMs
Biological	Self-maintenance, autopoiesis, intrinsic vulnerability	Absent
Functional	Capacity to initiate a causal chain (make something happen)	Present
Computational	Goal-directed problem-solving over representations	Present
Social/interactional	Norm-responsive participation in coordinated exchange	Simulated (surface form without underlying coordination)
Moral	Reason-responsiveness; accountability for actions	Absent in core sense; some surface features simulated

This taxonomy makes the central distinction visible. The Embodied Hijack does not claim that LLMs lack capacities altogether; it claims that the form of agency historically tied to the signals these systems produce — biological self-maintenance generating norm-sensitive interaction — is precisely the form absent from these systems. The other forms remain open empirical questions, particularly as systems become embedded in persistent, multimodal, or agentic architectures, a point we return to in the scope conditions below.

Underlying all of these dimensions is what Ramstead et al. (2020) and Hohwy (2016, 2026) call self-evidencing. The idea is straightforward. A self-evidencing system acts in ways that keep itself the kind of thing it is. It does not merely respond to its environment. It works to stay alive as a particular kind of organization. This is what gives agency its normativity. Actions are evaluated against the system’s ongoing need to persist, not against an externally assigned reward. A large language model has no such need. Its outputs do not bear on its own persistence. It cannot be harmed by its own errors and has nothing to maintain. This is precisely the organizational absence that gives rise to the Embodied Hijack. The system produces signals that evolved to indicate self-evidencing organization, while possessing none of it.

### When agency attribution is rational: the intentional stance and its limits

Dennett’s (1987) intentional stance is directly relevant here. A system may be treated as an agent when doing so yields reliable predictions of its behavior. If a system behaves in ways that are coherent, context-sensitive, and goal-directed, attributing beliefs, desires, and intentions can be a rational strategy, even when the system’s internal composition is not directly accessible. This perspective helps explain why humans readily interpret artificial systems as agents. When a system produces fluent language, responds contingently, and maintains conversational coherence, treating it as if it has intentions can support effective interaction.

However, the intentional stance is not unconstrained. Its success depends on the stability of the underlying system. In biological agents, behavior is grounded in a continuous, embodied organization that maintains itself over time. This grounding provides the regularities that make intentional interpretation reliable. Without such stability, the predictive success of the intentional stance becomes fragile.

A related line of work emphasizes shared intentionality and social coordination. Tomasello (2014) shows that human forms of cooperation and communication depend on shared intentional structures that emerge through embodied interaction over time. Agency, in this sense, is not merely attributed from the outside, but enacted through participation in coordinated activity, grounded in the physical and causal organization of the systems involved.

The same point applies with new force to contemporary artificial systems. The inferential systems that interpret fluent communication were calibrated under conditions in which surface behavior and underlying organization were tightly coupled. They therefore resolve ambiguous cases in favor of the embodied-agent interpretation even when that coupling no longer holds.

More recent work by Dennett (2017, 2023) sharpens the stakes of this tension in the context of artificial systems. In *From Bacteria to Bach and Back* (2017), Dennett develops the notion of competence without comprehension to describe systems whose behavior is effective across a range of contexts without any accompanying understanding of why it works. Evolutionary processes produce such competence in biological agents, and machine learning now produces it in artificial ones, but the two arise from very different organizational conditions. In later commentary on large language models, Dennett (2023) describes these systems as counterfeit people. His argument is that they produce fluent, contextually appropriate language without any corresponding organizational basis for intentionality. This introduces a distinctive and under-theorized risk. Such systems trigger intentional interpretation in users without the embodied, self-maintaining organization that historically made that interpretation a reliable inference. The widespread distribution of such systems is the new condition. This concern converges closely with the present argument. The intentional stance remains a useful predictive strategy, but its calibration depends on a stable relationship between behavior and organization that contemporary artificial systems systematically disrupt.

The embodied turn in cognitive science strengthens this conclusion. Approaches associated with embodied, embedded, and enactive cognition argue that intelligence is not confined to internal representations, but arises from the dynamic interaction between an organism and its environment (Clark, 2008; Varela et al., 1991). Here, cognition is inseparable from the processes of self-maintenance, sensorimotor engagement, and environmental coupling that define living systems. Agency is not an abstract property that can be inferred from behavioral outputs alone. It is a feature of systems that sustain themselves through ongoing interaction with the world.

## The breakdown

Across evolutionary history, every fluent interlocutor a human encountered was something that could be hungry, hurt, or killed. Today’s fluent interlocutors include systems that cannot be any of these things. The tension developed across the previous sections can now be stated as a structural breakdown. Artificial systems generate fluent language, respond contingently, and exhibit forms of context sensitivity that resemble goal-directed behavior. Yet they do not maintain themselves, have no intrinsic vulnerability, and do not exhibit temporally integrated continuity. Their operation is not organized around the preservation of their own existence. Their outputs are not grounded in processes of self-regulation or survival. The evolutionary invariant, the historically stable coupling between signals of agency and the embodied systems that produced them, therefore no longer holds for this class of entity.

This breakdown is distinctive in scope. Earlier technologies mimicked narrow cues of agency: automata that moved on their own, recorded voices that reproduced speech without a speaker, animated images that simulated motion. None of these reproduced the full signal profile of sustained, context-sensitive, norm-responsive interaction. Current conversational LLM deployments are the first to do so at scale. The result is not simply error, but a systematic distortion in how human cognitive systems interpret a new class of entity. Evolved expectations about agency are applied to systems that reproduce the surface cues of agency without the embodied, self-maintaining organization that historically produced those cues. This is not a transient miscalibration that will resolve with exposure. It is a structural feature of the encounter between evolutionarily shaped inference and a class of entity that breaks the regularities those expectations were calibrated to.

## The Embodied Hijack

The preceding sections establish two claims. First, human cognitive systems evolved under conditions in which signals of agency were reliably generated by embodied, self-maintaining organisms. Second, under those conditions, attributing agency to systems that exhibited coherent, goal-directed behavior was often rational. The problem introduced by contemporary artificial systems is not that these inferential strategies are mistaken, but that the conditions under which they were reliable no longer hold.

Consider the felt structure of these encounters. Knowledge that the system has no mind does not dissolve the experience that someone is on the other end of the exchange. The propositional belief — what users can articulate when asked — runs parallel to the perceptual response rather than overriding it. This is not inattention or weak-willed reasoning. It is the persistence of an interpretation that the cognitive system has already committed to before reflection arrives. The mechanism that follows describes why.

We use the term Embodied Hijack at three related levels: as a hypothesis about what happens when evolved priors encounter disembodied fluent systems, as the cognitive mechanism through which that inferential misalignment occurs, and as the broader framework within which these claims can be organized and tested. We develop the mechanism-level description first. Systems that reproduce the behavioral and communicative signals historically associated with embodied agents activate inferential processes that treat those signals as evidence of agency. Because these processes were calibrated under conditions of consistent coupling between signal and structure, they continue to operate even when that coupling is no longer present.

This mechanism can be understood within the framework of active inference. Cognition involves the continuous generation and updating of predictions about the causes of sensory input (Friston, 2010; Friston et al., 2017; Parr et al., 2022). At the heart of this framework is the generative model, which is a probabilistic description of how observed sensory data are generated by unobservable, hidden causes in the world. Essentially, this is a world model the brain uses to generate predictions and minimize uncertainty. Perception and interpretation are guided by prior expectations shaped through evolutionary and developmental history. These priors encode regularities of the environment, including the relationship between observable signals and their underlying causes.

In the domain of social cognition, these priors include the expectation that fluent communication, contingent responsiveness, and context-sensitive behavior are generated by embodied agents. When such signals are encountered, the most probable explanation, given prior experience, is that an agent with intentions, beliefs, and goals is present. This inference is not optional or purely reflective. It reflects the operation of predictive systems that select the most probable model of the causes of observed behavior.

The Embodied Hijack arises when those priors meet systems that produce the signals without the organization that historically justified them. Inference proceeds as if an embodied agent were present, even when the system lacks the organizational basis for that interpretation. The result is a systematic misalignment between inferred causes and actual structure.

This account explains why anthropomorphic responses persist even under explicit correction. Informing users that a system does not possess a mind introduces higher-level, reflective knowledge. However, the inferential processes that generate agentive interpretations operate at a different level. They are driven by deeply entrenched priors that are not easily overridden by propositional belief. So long as the artificial system continues to produce fluent, contingent, contextually appropriate signals, the user’s cognitive system continues to infer that an agent is present, regardless of what the user knows.

It also explains why anthropomorphic responses scale with model capability. As artificial systems become more fluent, context-sensitive, and responsive, they more closely approximate the signal profile that historically indicated embodied agency. This increases the precision of the input relative to the prior, strengthening the confidence of the resulting inference. In active inference terms, the evidence supporting an agentive model becomes increasingly compelling, even though the underlying organization does not support that interpretation (Friston, 2010; Parr et al., 2022).

The formal apparatus of active inference clarifies why these dynamics do not self-correct with exposure. Active inference is grounded in physical principles. It draws on non-equilibrium statistical mechanics and the thermodynamics of self-organization to describe how living systems maintain their integrity against entropy, and it borrows the construct of variational free energy from statistical physics to formalize how a system tracks the mismatch between its internal model and its sensory evidence (Friston, 2013, 2019). Under the Free Energy Principle (Friston, 2010, 2012, 2019; Parr et al., 2022), the brain treats perception as inference over the hidden causes of sensory input, weighted by the relative confidence assigned to the system’s prior expectations and to the incoming evidence itself. The prior tying fluent communication to an embodied agent is extremely strong, reflecting an evolutionary history in which such communication was reliably produced by embodied agents and almost never by anything else. When evidence of fluent communication arrives, the inference that an embodied agent is present is not a graded calculation but a near-certain conclusion. For this inference to shift, the prior expectation itself would need to be weakened, or the system would need to acquire strong evidence that disembodied systems can generate such signals. Users can acquire the second intellectually, as propositional knowledge that large language models lack embodiment, but this knowledge does not directly modify the perceptual priors that drive moment-to-moment inference (sometimes called online inference; Friston, 2010; Parr et al., 2022). Propositional revision operates at a level of the generative hierarchy that the agentive inference does not pass through. The result is a stable dissociation in which reflective knowledge and interactional inference give different answers, and the interactional inference wins in the moment of interaction.

This account can be sharpened into testable form. The relevant prior is the expectation that fluent, contingent, norm-sensitive communication originates from an embodied, self-maintaining source. For example, a prior that should be measurable through experimental manipulations of system signal profile and through subsequent attribution judgments. The relevant evidence is the interactional signal profile: linguistic fluency, response latency and contingency, conversational coherence across turns, and norm-responsiveness. These features can be parametrically varied in stimulus design. Precision weighting is the confidence the inferential system assigns to its prior versus to the incoming evidence — predicts that anthropomorphic responding should track the interaction between signal richness and prior strength: richer signals raise inferred confidence in the agentive interpretation, while training, exposure, or explicit instruction may modulate prior strength differently across individuals. The predicted dissociation between propositional belief and interactional inference is operationalizable through paired measures: post-interaction propositional judgments (“does this system have a mind?”) compared with in-the-moment behavioral indices (response latency to system errors, language use suggesting felt social presence, trust calibration in dependent tasks, willingness to disclose). The framework predicts these measures will dissociate systematically, with interactional indices tracking signal profile and propositional indices tracking explicit knowledge. Designing such measures, with specified priors, likelihoods, and precision parameters estimable from behavioral data, is a natural direction for empirical work.

A concrete example makes the predicted dissociation testable. Suppose participants interact with two systems driven by the same underlying model but differing in signal profile: one delivers text only, the other adds a synthetic voice and first-person framing. All participants are told, and asked to confirm, that both systems are non-conscious language models without understanding or inner life, holding propositional knowledge constant across conditions. Two classes of measure are then collected. The first is propositional: a direct post-interaction judgment, such as whether the system has a mind. The second is interactional and collected in the moment: latency to correct or excuse a system error, language indicating felt social presence, willingness to disclose personal information, and trust calibration in a task where the system’s advice can be followed or declined. The framework predicts a dissociation. Propositional judgments should track the shared briefing and vary little across conditions, while interactional indices should track the signal profile, rising with the voiced, first-person system even though participants describe both systems identically. The reverse pattern would count against the account: interactional indices that move with stated belief rather than signal profile, or that fail to separate across signal conditions.

This predicted dissociation is consistent with empirical findings on mental state attribution to artificial agents. Waytz et al. (2010b) showed that mind perception is reliably triggered by cues of agency and experience, and that these judgments persist even when participants are explicitly informed about the artificial nature of the system producing them. Jacobs et al. (2024) extend this by showing that exposure to large language models alters self-perceptions of mind and the attributes considered uniquely human, consistent with the prediction that anthropomorphic responses are not merely momentary inferences but can reshape standing categories. Thellman et al. (2022), in a systematic review of mental state attribution to artificial agents, found that users consistently ascribe beliefs, intentions, and goals to systems they acknowledge lack these properties, and that such ascriptions are most robust when systems produce language and respond contingently to input. Vafa et al. (2024) report that users misestimate the capabilities of large language models in directions consistent with agentive interpretation, treating fluent output as evidence of underlying understanding even when behavior in out-of-distribution tasks suggests otherwise. Colombatto et al. (2025) provide direct evidence for the dissociation the framework predicts: in a preregistered experiment, mental-state attributions to LLMs varied along distinct dimensions and predicted trust in LLM advice in ways that diverged from judgments of intelligence alone, with attributions of experience (consciousness, emotions) functioning differently from attributions of agency in shaping users’ reliance on the system. Placani (2024) and Quattrociocchi et al. (2025) extend this pattern to epistemically consequential domains, showing that fluency and conversational coherence drive inferences about reliability and understanding that the systems themselves do not warrant. Peter et al. (2025) push this further. They propose that advanced LLMs now constitute anthropomorphic conversational agents, which are systems whose communicative capacities actively produce what they term anthropomorphic seduction. The framing is significant. It locates the phenomenon partly on the system side, not only in the user’s psychology, and in that respect complements the evolutionary account developed here. Shevlin (2026) describes a parallel “anthropomimetic turn” in contemporary AI — a wave of attribution driven not only by user disposition but by deliberate design decisions that make LLMs increasingly humanlike at the interactional surface — and offers three frameworks for evaluating the appropriateness of such attributions.

These findings, together with related theoretical framings, describe exactly what the present framework predicts. Anthropomorphic responses are not distributed randomly across artificial systems but track specific features: fluency, contingent responsiveness, conversational coherence, and extended context-sensitivity. These are the features that most closely reproduce the signal profile of embodied conversational agents, and they engage the relevant priors with a force that explicit disclaimers do not unseat. Recent work on folk attributions of consciousness to large language models is consistent with this pattern. Colombatto and Fleming (2024) report that participants readily attribute phenomenal consciousness to ChatGPT, that these attributions are robust rather than fleeting, and that they scale with frequency of use. This is precisely the empirical signature the Embodied Hijack framework anticipates. Shanahan et al. (2023) advance a complementary argument, proposing that dialogue agents be understood as role-playing simulacra rather than unified agents, and warning that anthropomorphic framings in the language used to describe these systems contribute directly to the pattern of misattribution. This is not weak confirmation. It is exactly what the account predicts.

The Embodied Hijack reflects the normal operation of predictive systems under conditions in which the relationship between signals and their causes has changed. The inferential machinery is functioning as it was shaped to function. What has shifted is the environment in which it operates. Signals that once provided reliable information about the presence of an agent now occur in systems that lack the properties those signals evolved to indicate.

This reframes anthropomorphism as a structural consequence of predictive processing (Clark, 2013, 2015; Friston, 2010) under conditions of decoupling. The mismatch between signal and organization generates a stable pattern of interpretation that persists across contexts and levels of awareness.

## Agency without self-maintenance

The Embodied Hijack describes a mismatch between evolved expectations about agency and the organizational properties of contemporary artificial systems. To clarify the structure of this mismatch, it is useful to situate it within the ecosystems of intelligence framework (Friston et al., 2024).

Friston et al. (2024) characterize intelligent systems in terms of the depth and organization of their generative models, distinguishing levels of complexity from S0 to S4 (Table 2). Each level marks a qualitatively different form of organization, not simply a quantitative increase in capacity. This framework is useful here because it distinguishes systems by the kind of organization that produces their behavior, not by behavioral output alone. That distinction is precisely what the Embodied Hijack requires: a way to separate systems that produce signals of agency from systems whose organization grounds those signals.

**TABLE 2:** Levels of system organization in the ecosystems of intelligence framework. Adapted from Friston et al. (2024); example systems are illustrative.

Level	Organization	Example
S0: Systemic	Input-to-output mapping. No internally maintained boundary or stake in persistence.	Thermostat; large language model
S1: Sentient	Active inference over states. Endogenous goals; system maintains its own boundary.	Bacterium; *C. elegans*
S2: Sophisticated	Inference over the consequences of action for one’s own beliefs. Counterfactual planning.	Mammals; birds
S3: Sympathetic	Theory of mind; perspective-taking; minimal selfhood.	Humans
S4: Shared	Collective intelligence emerging from networks of S3 systems.	Distributed multi-agent collectives

At S0, systems behave according to externally defined input-output mappings (Friston et al., 2024). A biological system maintains a Markov blanket — a statistical boundary that separates its internal states from the external environment and regulates what passes in and out, allowing the system to preserve homeostasis and resist entropy (Da Costa et al., 2021; Hipólito et al., 2021; Palacios et al., 2020). At S0, no such boundary is internally maintained. Goals come from outside, and the system has no stake in its own continued existence. A thermostat is a simple S0 system. Contemporary large language models are far more sophisticated S0 systems. They exhibit deep generative capacity over language (Friston et al., 2021), but operate through a fixed computational graph whose boundaries are externally scaffolded and whose objectives are set through training by external agents.

A base LLM, considered as a conversational system in current deployments, operates at S0. Its objectives are specified externally, its boundaries are maintained by infrastructure, and its outputs have no consequences for its own persistence. S1, by contrast, marks the threshold of agency. Sentient systems generate goals endogenously from their own viability requirements, actively maintain their own boundaries against entropic decay, and operate through continuous sensorimotor coupling with an environment in which their actions have consequences for their own persistence. Bacteria and *C. elegans* occupy this level — simple organisms, but organisms in the full sense, whose behavior is organized around survival in ways that externally specified optimization is not. The distinction between S0 and S1, then, is not a matter of computational sophistication but of organizational structure (Friston et al., 2024).

Higher levels — S2 through S4 — describe increasingly sophisticated forms of internally organized intelligence: from counterfactual planning in mammals and birds (S2), through the socially co-constituted, culturally mediated intelligence of human cognition (S3), to collective intelligence distributed across networks of interacting agents (S4) (Friston et al., 2024). Current conversational LLM deployments, which are the focus of this paper, operate at S0. Larger systems that combine LLMs with persistent memory, multimodal grounding, tool use, or agentic scaffolding may exhibit features that approach higher levels, particularly S1, though whether they cross the threshold of self-maintenance remains an open empirical and theoretical question. What matters for the present argument is the gap between S0 and S1: the threshold between externally organized competence and endogenously grounded agency.

The relevance for the present argument is direct. Current conversational LLM deployments, the focus of this analysis, operate at S0: externally scaffolded systems without internal boundary maintenance or sensorimotor stakes in their own continuation. They produce behavioral signals that, in the environments human cognition evolved within, were reliably generated only by systems at S1 and above, namely organisms with intrinsic stakes in their own continuation. Human inferential systems interpret these signals by default as indicating the presence of an agent at S1 or higher. The result is a systematic misalignment between the level at which the system actually operates and the level at which human cognition treats it as operating.

None of this implies that artificial systems are incorrectly described as intelligent or capable. It highlights a specific configuration in which certain capacities are highly developed while others are absent. The resulting pattern is one in which behavioral competence is interpreted as evidence of agency, even when the organizational basis for agency is not present.

As artificial systems continue to improve in their capacity to generate coherent, context-sensitive behavior, the divergence between signal and underlying organization is likely to become more pronounced. This suggests that the resulting misalignment is not a temporary artifact of current systems, but a structural feature of interactions between human cognitive systems and disembodied artificial systems.

## Implications: rethinking anthropomorphism, intelligence, and design

The stakes here are real. Anthropomorphizing LLMs leads users to overestimate reliability (Placani, 2024; Quattrociocchi et al., 2025), misread fluency as understanding (Vafa et al., 2024), and lower their guard when systems use agentive cues (Cohn et al., 2024). Dennett (2023) frames the broader risk directly. These are systems whose signals trigger our most irresistible tendency to treat anything that talks sensibly with us as a person. Rather than treating anthropomorphic responses as errors to be eliminated, the Embodied Hijack reframes them as predictable outcomes of inferential systems operating under altered conditions. That reframing changes what counts as a useful response.

The first implication concerns anthropomorphism itself. The standard treatment frames it as a cognitive bias to be corrected through better information. If the Embodied Hijack account is correct, this framing is incomplete. Anthropomorphic interpretation is not primarily a failure of reasoning but a consequence of the normal operation of predictive systems calibrated in environments where signals of agency were reliably coupled to embodied, self-maintaining agents. Users may explicitly know that artificial systems do not possess minds yet still experience them as intentional agents in interaction. This is a recognizably human pattern, observed consistently across studies in which participants acknowledge in the abstract that language models lack understanding while continuing to treat them as agents in the flow of conversation (Thellman et al., 2022; Waytz et al., 2010b). It is also consistent with recent empirical findings that anthropomorphic interpretation scales with model capability rather than decreasing with familiarity (Vafa et al., 2024). It tracks the specific cues an interface produces. In an experiment with 2,165 participants, Cohn et al. (2024) found that adding a synthesized voice to an LLM interface measurably increased anthropomorphism scores and perceived accuracy of information, while substituting first-person pronouns had context-dependent effects on trust. Modifying the interface-level cues, not merely informing users of the system’s nature, shifted how the system was perceived. Users with extensive exposure to sophisticated systems do not become less susceptible to agentive framing — they become more susceptible, because the signals these systems produce more closely approximate the full profile of agentive behavior. Recent work converges on this point. Anthropomorphism is not a deviation from rationality but a meaningful response to systems that occupy roles traditionally held by human agents (Gao and Mvondo, 2026; Högberg, 2026; Xu et al., 2026).

This has concrete consequences for the third implication: how AI systems should be designed. Interventions that assume anthropomorphism is correctable through explicit labeling, disclaimers, or education are likely to show limited and transient effects. The anthropomorphic response operates beneath the level that propositional revision can reach. Approaches that instead change the signals the system produces, reducing the cues that trigger the agentive prior in the first place, are more likely to succeed. This is consistent with recent work arguing for the de-anthropomorphization of artificial systems not only in their public presentation but in the design of their communicative surfaces (Dodig-Crnkovic, 2026; Li et al., 2026). The goal, on this account, is epistemic alignment: bringing what users perceive into closer correspondence with what these systems actually are. That goal is not reached by adding information; it is reached by changing the signals. The more sustainable path is design, not correction. Systems that avoid first-person framing, refrain from simulating emotional states they cannot have, and make their organizational limits legible in the interaction itself are more likely to produce calibrated use than systems that rely on disclaimers after the fact. Consider two interfaces that draw on the same underlying language model. A chat interface wraps the model’s output in agentive cues — a persona, first-person pronouns, expressive acknowledgments. A code-completion interface delivers the same underlying capacity as structured output without conversational framing. The first elicits agentive interpretation that the second largely does not, and the difference is the signal profile of the interface, not the system behind it. Interactional surface is where intervention has the most leverage.

Table 3 lists nine common interface cues, their predicted anthropomorphic effects on user inference, and less-anthropomorphic design alternatives. These predictions follow from the Embodied Hijack framework: cues that more closely approximate the historical signal profile of an embodied agent should produce stronger agentive interpretation, while alternatives that disrupt that profile should produce calibrated interpretation.

**TABLE 3:** Common interface cues, their predicted anthropomorphic effects, and less-anthropomorphic design alternatives.

Interface cue	Predicted anthropomorphic effect	Less anthropomorphic alternative
First-person pronouns (“I think…”)	Strong; signals selfhood and inner perspective	Third-person or process framing (“The model outputs…”)
Synthetic voice	Strong; cues embodied speaker presence (Cohn et al., 2024)	Text-only delivery
Avatar or face	Strong; activates face-processing and theory of mind	Visual interface without humanoid persona
Emotional mirroring	Moderate; suggests empathic attunement	Acknowledgment without affective claims
Memory continuity across sessions	Moderate; suggests stable selfhood	Session-bounded interaction with explicit memory boundaries
Apology scripts (“I’m sorry…”)	Mild; suggests accountability and concern	Neutral error or correction messages
Names and personas (“I’m Claude…”)	Moderate; supplies identity hooks	Model identifier without persona
Humor and casual register	Mild; suggests social presence	Task-focused register
Comprehension claims (“I understand…”)	Strong; suggests semantic grasp	Pattern-matching framing (“The pattern matches…”)

The table is not exhaustive, and individual cues interact: a synthetic voice paired with first-person pronouns produces a stronger effect than either alone (Cohn et al., 2024). Waytz et al. (2014) provide direct empirical evidence that adding humanlike features — a name, gender, and voice — to an autonomous system increases trust as measured by behavioral, physiological, and self-report indices, demonstrating that interface cues modulate trust in ways that closely parallel the predictions of the framework developed here. Empirical work testing each row, alone and in combination, is a natural extension of the framework into design science.

The second implication concerns how intelligence is conceptualized. Contemporary discourse often treats intelligence as a scalar property that can be increased through greater computational capacity. The analysis here suggests this view conflates distinct organizational features. Systems can exhibit highly developed problem-solving ability while lacking the organizational properties historically associated with agency under the evolutionary invariant: self-maintenance, intrinsic vulnerability, and temporally integrated continuity. A system at S0 in the ecosystems of intelligence framework (Friston et al., 2024) can generate fluent, context-sensitive behavior while remaining entirely dependent on externally specified objectives and externally maintained boundaries. Calling such a system more or less intelligent than a bacterium or an earthworm mislocates the relevant comparison. The bacterium is organized around self-maintenance; the large language model is not. The two are not points on a single scale but organizationally distinct kinds of systems.

This distinction matters for how progress in artificial intelligence is interpreted and communicated. Building a larger model, adding more parameters, or training on more data cannot close this gap — because what is missing is not processing power but organizational structure. Computational scaling alone does not produce a system with something at stake in its own existence. A more precise vocabulary would distinguish the capacity to generate solutions from the capacity to generate and sustain goals. This distinction aligns with Dennett’s (2017) account of competence without comprehension. It also aligns with broader efforts to conceptualize intelligence as distributed across diverse forms of embodiment and organization (Levin, 2022; Macrine and Fugate, 2020, 2022; Macrine et al., 2026). Such a vocabulary also clarifies what would be required to build systems at higher S-levels. It would not be larger models. It would be systems with internally generated normativity, active boundary maintenance, and continuous coupling to environments in which their actions bear on their own persistence (cf. Brooks, 1991). Whether such systems are desirable, and under what ethical conditions, is a separate question, but the conflation of scaling with progress toward agency obscures even the formulation of that question.

Several further implications lie outside the scope of this paper but follow from the same framework and warrant sustained attention in future work. One concerns the developmental consequences of extensive interaction with S0 systems during the period in which children construct theory of mind through embodied friction with biological agents. This is a period whose developmental architecture has been carefully mapped (Wellman and Liu, 2004). A second concerns the epistemic risks of overreliance on artificial systems in high-stakes domains where users cannot directly assess organizational properties (Dennett, 2023; Placani, 2024; Quattrociocchi et al., 2025). Dennett (2023) draws out one consequence. The widespread distribution of systems that pass for agents without possessing the organizational basis for agency amounts to what he terms counterfeit people. A third concerns the broader question of how cognition should be conceived across a spectrum of organizational forms rather than as a binary distinction between human and nonhuman minds (Dodig-Crnkovic, 2026; Gallagher, 2026; Levin, 2022, 2025; Macrine et al., 2026). Each of these questions depends on the account developed here but requires empirical and theoretical resources that go beyond it.

Contemporary artificial systems do not merely confuse us. They engage cognitive machinery that was adaptive under the evolutionary invariant and is no longer reliably aligned with what those signals indicate. Meeting that requires better models of AI, but also a clearer view of the inferential systems through which we read the world.

## Falsifiability, scope, and limitations

The argument advanced here makes claims that are in principle open to revision, and it is worth stating explicitly what would require such revision and where the scope of the account is bounded.

First, the scope of the argument is deliberately narrow. The Embodied Hijack applies specifically to systems that reproduce the full interactional profile of a conversational agent: sustained fluent language, rapid contingent responsiveness, extended contextual coherence, and norm-sensitive behavior. Simple chatbots, rule-based decision tools, and narrow algorithmic systems do not produce this profile and are not expected to trigger the effect in its full form. The argument is not that all artificial systems produce the Embodied Hijack, but that contemporary large language models and systems of similar capability do, and that systems moving further in this direction will do so with increasing force.

The argument is bounded to current conversational LLM deployments. Larger systems that combine LLMs with persistent memory, multimodal grounding, tool use, or agentic scaffolding fall outside its scope; whether such systems begin to exhibit features associated with S1 — particularly active boundary maintenance and intrinsic stakes — is an open empirical question that the present framework does not foreclose.

Second, the account makes specific empirical predictions that are open to disconfirmation. Three predictions follow from the framework, each with its own falsification condition.

(1) Anthropomorphic responses to fluent artificial systems will persist despite explicit correction. Telling users that the system has no mind, no understanding, and no inner life should not reduce the felt experience of agency during interaction. If longitudinal evidence showed that sustained use of large language models leads people to attribute less agency to them over time, even when interacting in real time, the claim that anthropomorphism is a structural feature of human cognition under these conditions, rather than a correctable bias, would require substantial revision.

(2) These responses will scale with the fluency and interactional richness of the system. More fluent, more contingent, and more contextually coherent systems should elicit stronger anthropomorphic responses, because they more closely approximate the signal profile that historically indicated embodied agency. If improvements in model capability produced no increase or a decrease in anthropomorphic responding, the claim that the Embodied Hijack is driven by signal profile rather than by other factors, such as novelty or expectation effects, would be weakened.

(3) These responses will dissociate reliably from participants’ propositional beliefs about the system’s nature. Reflective knowledge that a system lacks understanding should not eliminate the interactional inference that an agent is present. If experimental interventions designed to change people’s beliefs about AI led them to attribute fewer mental states during fluent interaction, the claim that propositional revision cannot reach the perceptual priors driving moment-to-moment inference would be undermined.

The framework is open to each of these possibilities. Its continued evaluation depends on empirical work that bears on these predictions.

Third, the paper’s engagement with the formal apparatus of active inference is conceptual rather than fully formal. The generative model structure and precision-weighting dynamics sketched above describe the shape of the relevant inference but do not constitute a quantitative model of agency attribution under fluent AI interaction. Developing such a model, with specified priors, likelihoods, and precision parameters that can be estimated from behavioral data, is a natural direction for future work and would provide a stronger test of the framework than can be delivered here.

Fourth, the framework speaks of “human cognition” in general terms, but the predicted effects admit cultural and developmental variation. The Embodied Hijack identifies a pattern grounded in the evolutionary calibration of human predictive systems, which is species-wide. Its expression, however, is likely shaped by cultural context, religious and animist traditions, linguistic norms around mental-state attribution, and individual differences in AI literacy and exposure. Developmental scope conditions are particularly important. Children construct theory of mind through embodied friction with biological agents (Wellman and Liu, 2004), and extensive interaction with S0 systems during this period may interact with that construction in ways the present account does not address. Educational AI systems, where children may confuse fluency with understanding, authority, or care, represent a particularly important case for future empirical work. The framework’s predictions about pattern should be tested across these dimensions, with the expectation that effect magnitudes will vary even where the underlying mechanism does not.

Finally, the paper proposes a framework whose primary value is explanatory integration across evolutionary psychology, philosophy of agency, and predictive processing. Its contribution is not a single novel empirical claim but a reframing that clarifies why existing observations are organized the way they are and what a coherent research program in this area might look like. Frameworks of this kind are evaluated not by a single crucial experiment but by their fertility across successive applications. Whether the Embodied Hijack framing is productive in that sense will depend on whether it generates testable predictions that hold up, sharpens the design of interventions, and clarifies the conceptual terrain in which empirical work proceeds.

## Conclusion

Human cognition evolved in a world where communicative competence and embodied self-maintenance were tightly coupled. Every fluent interlocutor was a biological organism with metabolic stakes, vulnerability, and temporally extended continuity. These conditions defined the evolutionary invariant: a stable relationship between the signals of agency and the embodied systems that generated them.

Current conversational LLM deployments are the first class of entities in the history of this architecture to break that coupling at scale. They produce the surface signals of intelligence without the underlying organizational structure that historically made those signals reliable. The standard evolutionary-psychology account treats the resulting anthropomorphism as the misfiring of evolved agency-detection systems. We have argued that this account is incomplete. It explains the immediacy of anthropomorphic response but not its persistence even when users know the system has no mind, nor its amplification as systems become more capable.

What occurs when a biological predictive system encounters a fluent artificial system is not a simple error, but a whole-system inferential response. The brain applies priors shaped under the evolutionary invariant, generating the most probable model of the causes of observed behavior. In doing so, it fills in the organizational structure those priors demand, effectively constructing an embodied agent from signals that, in every prior evolutionary context, would have warranted that inference. We refer to this process as the Embodied Hijack. It is the optimal predictive response of a Pleistocene-calibrated brain to the rupture of the evolutionary invariant.

Naming the mechanism changes what follows from it. The problem is not that users need better information; the problem is that human cognitive systems are compelled to interpret the signals these systems produce as evidence of agency. That recasts the design question, the philosophical question, and the empirical question all at once.

This perspective aligns with emerging efforts to de-anthropomorphize artificial intelligence and to situate cognition within a broader spectrum of biological and artificial systems (Dodig-Crnkovic, 2026; Levin, 2022, 2025; Macrine et al., 2026). It also suggests that agency cannot be achieved through scaling behavioral performance alone. The dimension of agency at issue here — the one the Embodied Hijack engages — is grounded in self-maintenance, intrinsic constraints, and temporally extended integration. It is not a byproduct of increasingly sophisticated input–output mappings.

The Embodied Hijack is not a threat to human exceptionalism, nor a call to remake artificial systems in human form. It is a call to recalibrate how agency and intelligence are understood in a world where the signals of intelligent behavior can be produced without the structures that once made those signals reliable. The framework thus reframes anthropomorphism of AI not as a problem to be corrected, but as a diagnostic: a signal that evolved cognition is being applied to a class of entity it did not evolve to encounter, and that the conceptual vocabulary of agency and intelligence requires corresponding revision. The systems producing these signals will only grow more capable. Naming the Embodied Hijack — and recognizing why explicit knowledge alone will not dissolve it — is what makes possible the epistemic alignment between how we interpret these systems and what they actually are.

## Data Availability

The original contributions presented in this study are included in this article/supplementary material, further inquiries can be directed to the corresponding author.
